# A Study of the Diversity Patterns of Desert Vegetation Communities in an Arid Zone of China

**DOI:** 10.3390/plants13192783

**Published:** 2024-10-04

**Authors:** Zhiming Xin, Xing Li, Yonghua Li, Xue Dong, Ruibing Duan, Xu Chang, Yiben Cheng, Xiuqing Wu, Wei Li

**Affiliations:** 1 School of Soil and Water Conservation, Beijing Forestry University, Beijing 100083, China; xinzhiming@caf.ac.cn; 2 Experimental Center of Desert Forestry, Chinese Academy of Forestry, Dengkou 015200, China; lixing19942022@163.com (X.L.); dongxue98765@126.com (X.D.); duanruibing@caf.ac.cn (R.D.); 3 Inner Mongolia Dengkou Desert Ecosystem National Observation Research Station, National Forestry and Grassland Administration, Dengkou 015200, China; 4 Institute of Ecological Conservation and Restoration, Chinese Academy of Forestry, Beijing 100091, China; lyhids@caf.ac.cn (Y.L.); liwei@caf.ac.cn (W.L.); 5 Applied Geological Research Center, China Geological Survey, Chengdu 610036, China; xzmlkn@163.com

**Keywords:** plant community, β-diversity, environmental filtering, species turnover, climate change

## Abstract

The Gobi Desert ecosystem is currently experiencing the impacts of persistent climate warming and extreme weather. However, the relative influences of factors such as soil, climate, and spatial variables on the β-diversity of desert plants and their key components have not been systematically studied. In this research, the Dunhuang North Mountain and Mazong Mountain areas were selected as study areas, with a total of 79 plant community plots systematically established. The aim was to explore intercommunity β-diversity and its components and to analyze the interrelationships with climate factors, soil factors, and geographic distance. The results indicate that (1) there is a geographic decay pattern and significant differences among plant communities in the Dunhuang North Mountain and Mazong Mountain areas, with β-diversity primarily driven by replacement components. (2) Climate, soil, and geographic distance significantly influence β-diversity and its replacement components, with climate factors exerting the greatest influence and geographic distance the least. (3) Multiple regression analysis (MRM) reveals differential effects of climate factors, soil factors, and geographic distance on β-diversity and its replacement components, with climate and soil factors exerting a much greater influence than geographic distance. In summary, the β-diversity of plant communities and their replacement components in the Dunhuang North Mountain and Mazong Mountain areas result from the combined effects of habitat filtering and dispersal limitation, with habitat filtering having a greater impact, while environmental heterogeneity is an important factor influencing species differences in this region.

## 1. Introduction

Drylands cover nearly 41.5% of the Earth’s land surface and are the single most extensive form of land on the planet [1]. Dryland ecosystems are highly vulnerable, biodiversity conservation is an important way to build a better ecological environment and maintain sustainable development, especially in the Gobi Desert [2,3]. Biodiversity is categorized into α-diversity, β-diversity, and γ-diversity, with β-diversity representing differences in species composition among different communities, acting as a link between α and γ-diversity [4]. Studying β-diversity not only helps understand the relationship between species and the environment but also sheds light on the ecological processes driving community assembly [5,6]. The pattern of β-diversity is generally considered to be determined by the combined effects of environmental filtering, dispersal limitation, and other unknown historical processes [7]. Ecological niche theory posits that community assembly is governed by deterministic processes such as niche differentiation, while neutral theory suggests that community assembly is dominated by stochastic processes such as drift, dispersal, and speciation [8,9]. Researchers have systematically integrated niche theory and neutral theory, suggesting that both deterministic and stochastic processes are crucial for the formation of community β-diversity, albeit with differing degrees of importance. β-diversity can further be decomposed into species turnover (or replacement) and nestedness (or richness difference) components [10,11,12]. Species turnover represents the replacement of species along spatial or environmental gradients, driven by mechanisms including environmental filtering, competition, and geographic isolation [13,14,15]. Nestedness represents the gaining or losing of species along specific environmental gradients, driven by the-diversity of ecological niches and ecological processes such as selective extinction, selective immigration, and habitat nesting [16]. Deconstructing β-diversity into turnover and nestedness components, and investigating the roles of these components in overall β-diversity and their spatial distribution patterns [17,18], facilitates our understanding of the underlying processes shaping plant community-diversity, enhances our understanding of biogeography and ecological issues [19], and holds significance for biodiversity conservation and ecosystem management.

Environmental and spatial processes are key factors influencing community assembly, with habitat filtering and dispersal limitation playing important roles in the formation of β-diversity [20], processes associated with niche theory and neutral theory, respectively. Niche theory posits that environmental factors determine species coexistence, with each species having its own adapted environment [21]; as habitat heterogeneity increases, species composition generally becomes more dissimilar, spatially corresponding to habitat similarity [22]. Climate, as a crucial environmental factor, has long been a focal point in studies of its effect on species β-diversity [23]; additionally, soil factors are important in influencing vegetation characteristics. Neutral theory suggests that random dispersal is one of the key mechanisms of community assembly, with the dispersal ability diminishing with the increasing geographic distance [24]; thus, geographic distance is commonly used to measure dispersal limitation. Numerous studies have confirmed the significant impact of geographic distance on community β-diversity [25,26,27,28]. Furthermore, elevation, by potentially increasing the heterogeneity of environmental factors, including climate, and increasing the spatial isolation of species, may enhance community dissimilarity [29]. With further research, it has been increasingly demonstrated that the combined effects of habitat filtering and dispersal limitation promote the formation of β-diversity patterns.

The Gobi Desert habitat exhibits considerable spatial heterogeneity in factors such as geology, topography, and climate, giving rise to unique plant resources [30]. It has a unique combination of aridity, extreme climate, geographic location, and ecological gradients. The plant community structure in the Gobi Desert is simple, dominated by shrubs, with a sparse herbaceous layer. The Gobi Desert is in the temperate area of China, with a tough environment, infertile soil, impoverished community types, and a single species life type, mainly dominated by temperate desert shrubs and half-shrub communities and forming temperate scrubs and meadows in some areas. Compared to other desert areas, in terms of community species composition, the proportion of shrubs, half-shrubs, and small shrubs is higher; the soil and water conditions in the territory are more severe; the composition of communities is simpler; the dominant communities have a higher degree of predominance; and the composition of communities usually has two to five species; most of the community species are shrubs and half-shrubs, and there lacks short-lived plants in comparison with the composition of the desert vegetation. Current research on the Gobi Desert is relatively limited, primarily focusing on the characteristics and biodiversity of plant communities [31], with less attention given to changes in species diversity along elevation gradients within shrub communities. Studies specifically addressing β-diversity and its influence on the Gobi Desert vegetation is scarce. While there have been studies on the β-diversity patterns of plant communities in temperate desert regions of China [32], there is a lack of systematic research analyzing the relative impacts of β-diversity and its key components in the Dunhuang Gobi Desert. This study aims to address the following questions through vegetation surveys in the Dunhuang North Mountain and Mazong Mountain areas, coupled with data collection on climate, soil, and geographic distance: (1) What are the spatial distribution patterns of β-diversity and its components in the Dunhuang Gobi area? (2) How do geographic distance and environmental factors influence β-diversity and its components in plant communities? (3) What are the differences in the relative importance of geographic distance and environmental factors in driving the formation of β-diversity and its components patterns in plant communities? It is hoped that this research will provide insights into the patterns of β-diversity in plant communities in the Dunhuang Gobi Desert and the potential processes and influencing factors of β-diversity formation in arid zone plant communities.

## 2. Study Area

The study area was located in the West Gobi Desert, and the geographical location was between 92°09′~100°20′ E and 37°58′~42°48′ N, with an altitude range of 800~3200 m. The area is at the junction of the Inner Mongolia Plateau and Qinghai–Tibet Plateau and at the intersection of the Qinghai–Tibet Plateau biota and Central Asian biota. The main land types consist of oasis, wetlands, desert, and Gobi vegetation [33]. The annual precipitation amount in the study area varies greatly, with a maximum annual precipitation of 200 mm in Arjinshan and only 39 mm in Maxian Mountain. The annual potential evaporation is approximately 2000~4000 mm, the temperature difference between day and night is large, and the average annual temperature is 4~10 °C. The solar radiation is strong, and the total annual sunshine duration exceeds 4000 h. The primary soil type is brown desert soil. The dominant plant species are shrubs such as *Nitraria sphaerocarpa*, *Sympegma regalia*, and *Alhagi sparsifolia*.

## 3. Materials and Methods

### 3.1. Sample Plot Setting and In-Site Survey

**Figure 1:**
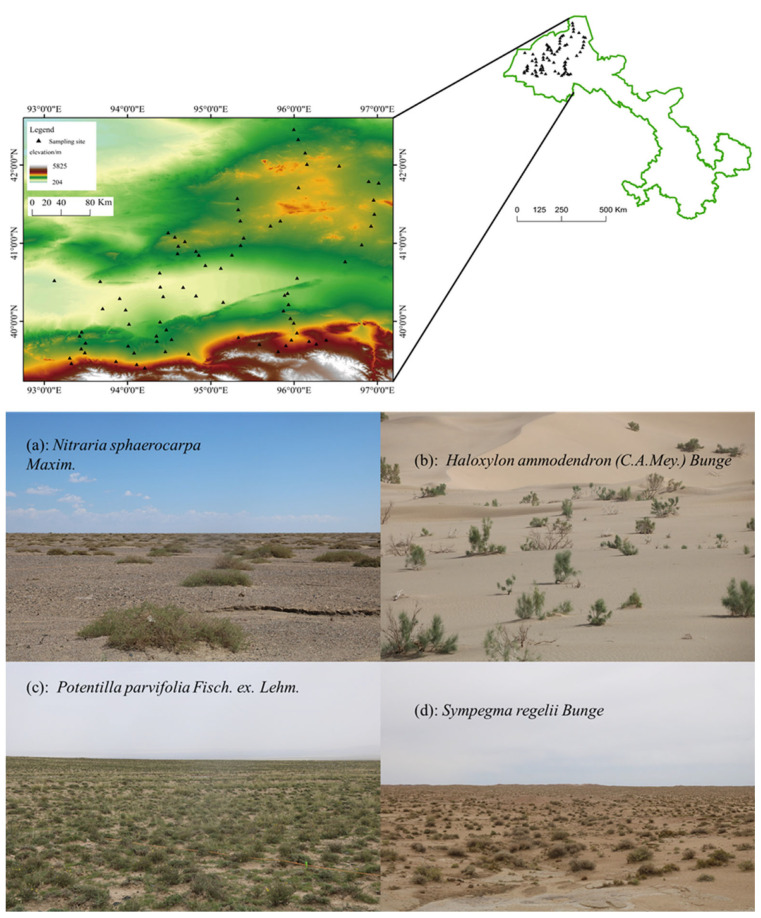
Distribution of sampling points and study area and vegetation communities in the study area. (**a**) *Nitraria sphaerocarpa* Maxim, (**b**) *Haloxylon ammodendron* (C.A.Mey.) Bunge, (**c**) *Potentilla parvifolia* Fisch. ex. Lehm, and (**d**) *Sympegma regelii* Bunge.

In August 2018 and June 2019, a sample survey of distinct plant communities in the study desert area was conducted, and one 100 m × 100 m sample plot was set up at each survey site. One 10 m × 10 m shrub sample was established at each of the four vertices and at the center of each sample plot, and the species names, heights, and canopy widths of all shrubs in each sample plot were determined and recorded. One 1 m × 1 m small sample plot was set up at each of the vertices, at the center, and in the middle of each vertex to the center of the sample plot for an herbaceous plant survey, and the species names, heights, multiplicity, and clump widths of all herbs were determined and recorded. In this study, at 79 sample plots, 395 shrub samples and 711 herbaceous samples were collected, as shown in Table 1. The distribution of the sampling points is shown in Figure 1.

### 3.2. Soil Survey and Sampling

In each sample plot at the survey site, we selected three shrub sample plots at random, and we chose three sampling points in each plot to obtain 0–10 cm depth soil samples with a soil auger. We mixed the soil from these three sampling points well and took 200× *g* for the determination of the soil physical and chemical properties. We repeated the sample collection three times. The mixed soil samples were brought back to the Desert Forestry Experimental Center of the Chinese Academy of Forestry laboratory for air-drying and sieving through a 2 mm soil sieve. The soil samples were analyzed for the following indicators: soil pH (water—soil ratio of 2.5:1), soil total phosphorus (STP), soil organic matter (SOM), soil total nitrogen (STN), and soil total potassium (STK).

### 3.3. Climate Data Acquisition

The climate data in this research were obtained from the World Climate Data website (https://www.worldclim.org/, accessed on 5 August 2024), and climatic factors were extracted based on the sample site’s latitude and longitude coordinates in the R language raster package to determine the raster geographical patterns of the sampling points, including the mean annual temperature (MAT), the monthly maximum warmest temperature (MTWM), the minimum temperature of the coldest month (MTCM), mean annual precipitation (MAP), precipitation of the wettest month (PWM), precipitation of the driest month (PDM), precipitation of the wettest season (PWS), and precipitation of the driest season (PDS).

### 3.4. Data Analysis

#### 3.4.1. Calculation of Bray–Curtis Dissimilarity Index

The Bray–Curtis distance index can characterize the difference in species composition between different plots. The calculation considers the presence or absence of species and the abundance of species. The similarity of the community assembly was calculated as the 1-Bray–Curtis distance index, and a linear regression of distance attenuation was performed by using community similarity transformed by ln (x) and the geographical distance transformed by ln (x) [34]. The slope of the distance attenuation curve reflects the species turnover rate. Using the ‘betapart’ toolkit in R, β-diversity was divided into two parts, turnover (*β_sim_*) and nestedness (*β_nes_*), to explore the effects of these two processes on species composition differences between communities. The Bray–Curtis distance index calculation equations are shown in Equations (1)–(4):

(1) $$Dbray=\frac{\sum_{i=1}^{p}\left|y_{ij}-y_{ik}\right|}{\sum_{i=1}^{p}\left|y_{ij}+y_{ik}\right|}$$

(2) $$\beta_{BC}=\frac{b+c}{2a+b+c}$$

(3) $$\beta_{sim}=\frac{\min\left(b,c\right)}{a+\min\left(b,c\right)}$$

(4) $$\beta_{nes}=\beta sor-\beta sim$$

where *p* is the number of species (number of species in the sample–species matrix), and *y_ij_* and *y_ik_* denote the corresponding species multiplicity in the two samples, *a* is the number of co-occurring species in the community, and *b* and *c* are the numbers of endemic species in the community.

#### 3.4.2. Environmental Distance Calculation

The ‘geosphere’ package was used to calculate geographical distance based on the latitude and longitude coordinates of the survey points, and then, the built-in function scale was used to standardize the environmental factors to eliminate dimensional differences. The ‘vegan’ package was used to calculate the Euclidean distance [35].

#### 3.4.3. Mantel Test

The correlation between geographical distance and environmental distance and β-diversity and its components was explored by the Mantel test. The partial Mantel test was used to examine the effects of spatial or environmental factors alone on plant community β-diversity and turnover components by controlling for environmental or geographical factors. For each test, the Spearman method was used, and 1000 random permutations were repeated to obtain the correlation r value and significance.

#### 3.4.4. Analysis of Multiple Regressions on Dissimilarity Matrices

To avoid the influence of multicollinearity, the ‘varclus’ function in the ‘Hmisc’ package was used to perform cluster analysis before matrix-based multiple regression on dissimilarity matrices (MRM) to evaluate the redundancy of environmental variables. Finally, altitude, total nitrogen, organic carbon, soil pH, annual average temperature, mean temperature of the coldest month, daily range of temperature, annual range of temperature, and geographical distance were retained for MRM analysis. The MRM function in the ‘ecodist’ package was used to test the significance of the environmental factors, and the residual significant variable matrix was used to fit the second regression to explore the effects of environmental factors and geographical distance on β-diversity. The factors were divided into two parts: geographical distance and environmental factors. The ‘varpart’ function in the ‘vegan’ package was used to evaluate the relative contribution of geographical distance and environmental factors. In addition, partial regression analysis was performed by MRM to further determine the contribution of diffusion limitation and environmental filtering to community assembly. All calculations and figures were processed in R4.1.2.

## 4. Results and Analysis

### 4.1. β-Diversity and Its Components in Plant Communities

**Figure 2:**
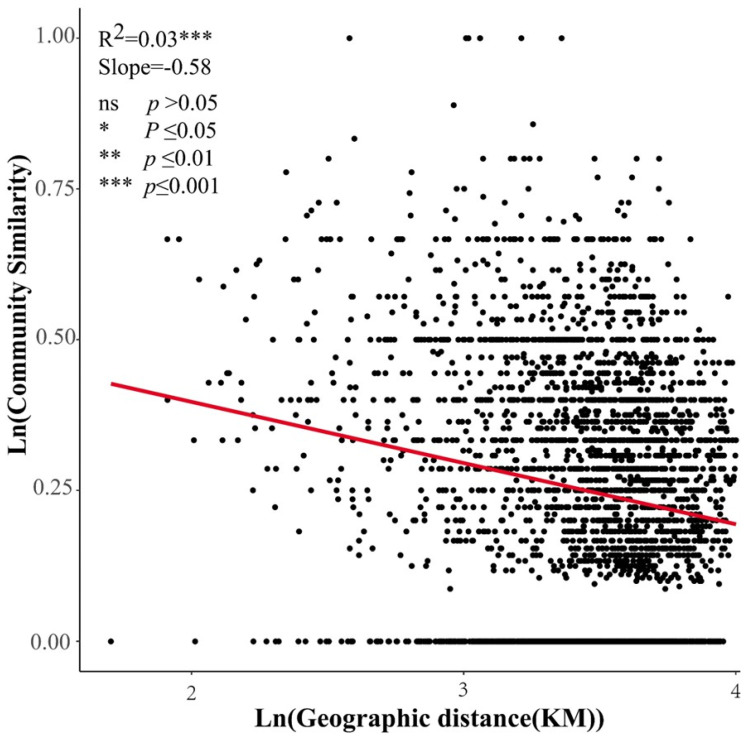
Decay relationship with geographical distance for plant community species in the study area.

The analysis of the sample survey from 79 sample plots in the study area yielded 57 species of seed plants belonging to 16 families and 45 genera. The proportion of species like Chenopodiaceae, Asteraceae, and Poaceae was high, accounting for 24.56%, 21.00%, and 12.28% of the total number of species, respectively. This result is shown in Figure 1. The total number of shrub and semi-shrub species was 29, and 19 species of perennial herbs and 8 species of annual herbs were found. The plant communities in the study area were dominated by shrubs, semi-shrubs, and perennial herbs. The relationship between intercommunity similarity and geographical patterns was analyzed by logarithmic transformation. An apparent decay relationship existed with geographical distance for plant community species similarity in the survey area (geographical patterns), with a sloping curve of 0.58 (absolute value), as shown in Figure 2, indicating that there was distinct species turnover in the plant communities of this area.

**Figure 3:**
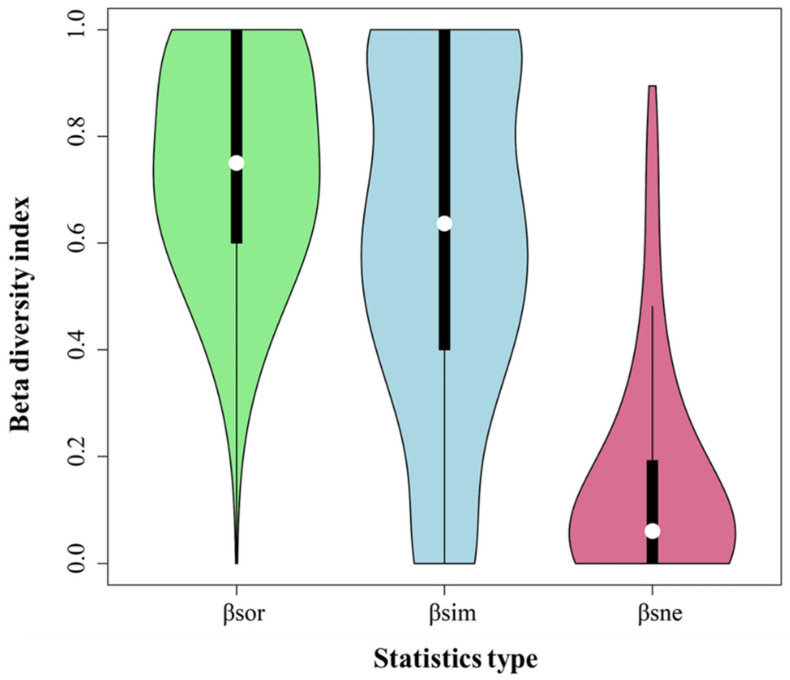
*β_BC_*, *β_sim_*, and *β_nes_* statistics based on the Bray–Curtis exponential algorithm.

The results of the Bray–Curtis distance index showed that the β-diversity index remained approximately 0.75, as shown in Figure 3, indicating that the similarity between the surveyed sample sites was low and that the species composition varied greatly. After deconstructing the β-diversity index into turnover and nestedness components, we found that turnover accounted for a large proportion and nestedness accounted for a small proportion. This indicated that the community turnover component was more critical than the nestedness component of β-diversity in the study area. The β-diversity of plant communities may mainly originate from community turnover. We focused on the relationship between the environmental factors and the turnover component.

**Figure 4:**
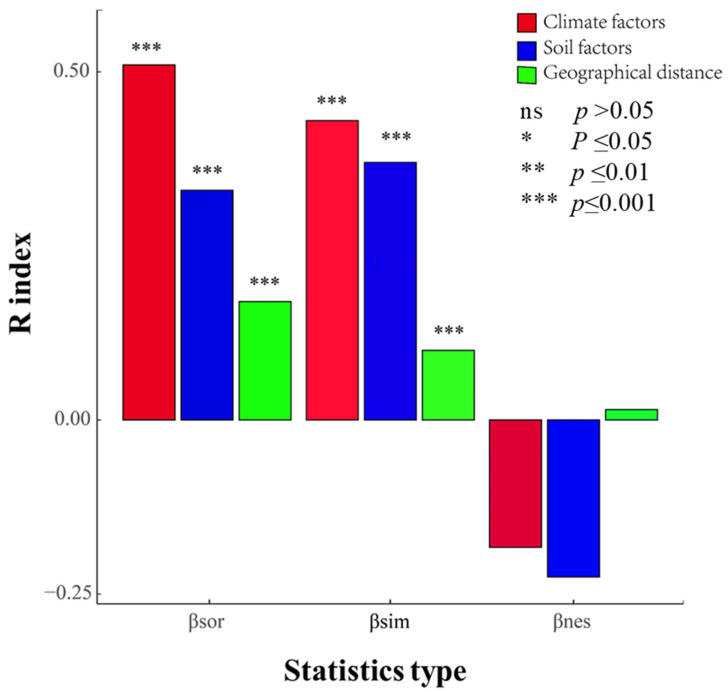
Mantel test of climate, soil, and geographical distance on β-diversity of nestedness and turnover. *** indicates significance at the *p* < 0.001 level.

We tested the effects of climatic factors, soil factors, and geographical distance on the β-diversity of vegetation communities and its turnover component based on the Mantel test. The results showed that vegetation community β-diversity and species turnover were significantly and positively correlated with climatic factors, soil factors, and geographical distance, as shown in Figure 4. As the differences in climatic factors, soil factors, and geographical patterns grew, the differences in species composition among communities became more prominent. The β-diversity of plant communities increased, and species turnover was consistent with the changes in β-diversity. The effects of climatic factors, soil factors, and geographical patterns on plant community β-diversity at the western end of the Hexi Corridor mainly arose from their effects on intercommunity species turnover, and climatic factors and soil factors had a more significant impact than geographical patterns. Climate and soil factors are the most important factors impacting the community dynamics in arid zones.

### 4.2. Effects of Environmental Factors and Geographical Distance on β-Diversity and Its Components

**Figure 5:**
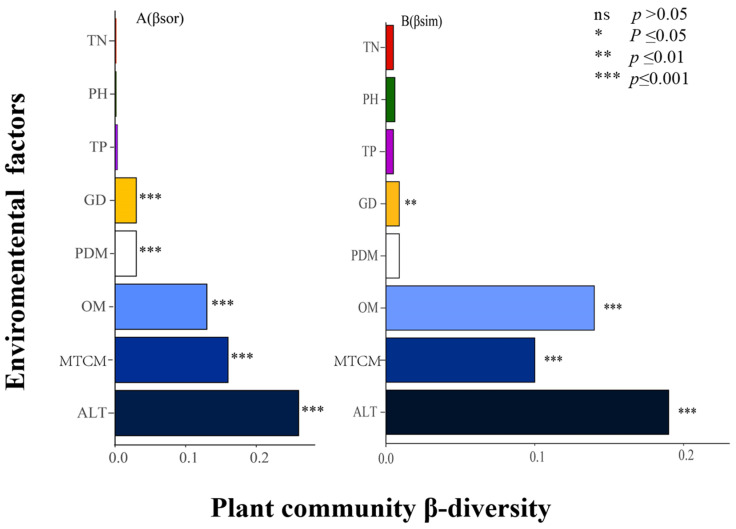
Effects of environmental factors and geographical distance on β-diversity and its turnover component. *β_sor_* and *β_sim_* represent β-diversity and turnover, respectively. *** indicates significance at the *p* < 0.001 level.

Environmental factor screening was performed with the various functions in the Hmisc package in R to avoid effects from multicollinearity. The screened environmental factors included minimum temperature in the coldest month (MTCH), precipitation in the driest month (PDM), altitude (ALT), total soil nitrogen (STN), total soil phosphorus (STP), soil organic matter (SOM), and soil acidity and alkalinity (pH). The interpretation of environmental factors and geographical distance and its components was carried out based on MRM. Climatic factors, as characterized by altitude and minimum temperature in the coldest month, and soil factors, as characterized by soil organic matter, had significant effects on plant community β-diversity. Dispersal limitation, as characterized by geographical distance, affected community β-diversity, but its effect was lower than that of climate and soil, as shown in Figure 5. In general, climatic and soil factors had more significant influences on plant community β-diversity, while geographical distance had a relatively minor impact on plant species turnover. A more detailed analysis showed that organic matter, extreme low temperature, and altitude were the three most important factors influencing community dynamics.

### 4.3. Roles of Climate, Soil Factors, and Geographical Distance on Community β-Diversity and Species Turnover

We analyzed the effects of climatic factors, soil factors, and geographical distance on β-diversity and its turnover components based on MRM, and they explained 27.0%, 13%, and 3% of the β-diversity, respectively. Climatic factors and soil factors together explained 32.0%, climatic factors and geographical distance together explained 17.0%, geographical distance and soil factors together explained 16.0%, and the three together explained 33.0% of the plant community dynamics. Climatic factors, soil factors, and geographical distance explained 19.0%, 15.0%, and 1% of the species turnover, respectively. Climatic factors and soil factors together explained 26% of the plant community dynamics. Climatic factors and geographical distance together explained 10.0% of the plant community dynamics. Geographical distance and soil factors together explained 15.0% of the plant community dynamics, and all three factors together explained 26% of the plant community dynamics, as shown in Table 2. This indicates that the environmental filters characterized by climatic and soil factors dominated the formation of β-diversity in the Gobi plant communities at the western end of the Hexi Corridor, and the dispersal constraints characterized by geographical patterns also played a specific role. These two factors together contributed to the desert community assembly.

## 5. Discussion

### 5.1. Community β-Diversity and Its Components in the Arid Zone

Community β-diversity indicates variations in plant communities and the degree of community differentiation associated with environmental gradients or patterns [36]. Our survey of plant communities in the North Mountain and Maxian Mountain areas of Dunhuang, as characterized by the Bray–Curtis distance index for β-diversity, showed that the plant communities in the area were highly variable (mean value 0.75). To quantify the community β-diversity, we decomposed it into two parts, turnover and nestedness, and analyzed the role of the two processes on β-diversity to understand the arid zone community assembly. The decomposition of β-diversity into turnover and nestedness revealed a species turnover ratio, which is consistent with a study conducted in the Kumtag Desert [37]. This indicates that species turnover may generate the β-diversity of plant community species [38]. In addition, in this study, we found that community species similarity decreased significantly with increasing the geographical distance, and a clear pattern of geographic decay was observed, similar to other studies [39,40]. Our present study revealed that the β-diversity of plant communities was influenced by climate and soil factors.

### 5.2. Effects of Climatic Factors, Soil Factors, and Geographical Distance on β-Diversity and Its Components

The influence of climate as a critical factor on community species turnover has been confirmed in numerous studies [41,42,43]. This study shows that the coldest month minimum temperature explained 16.0% and 10.0% of the β-diversity and turnover, respectively, as shown in Figure 4. Altitude contains combined factors that significantly influence spatial distribution patterns [44,45]. Altitude is a factor that reflects precipitation, radiation, and temperature [46]. Researchers found that altitude explained 18.9% of the β-diversity and 19.0% of the turnover of plant communities on the southern edge of the Kumtag Desert [47]. The maximum difference in altitude among the 79 sample plots in this study was 2353 m, which explained 29.0% of the β-diversity and 18.8% of the turnover component. This result showed high reliability for the species of plant communities in this study area, and the differences in altitude increased species turnover. This may have also been related to the altitude of the study area, which is high in the south and low in the north, sloping from southwest to northeast, and to the vertical divergence of climate with increasing altitude. Studies have shown that shrub communities in arid zones are not uniformly established across altitude gradients [48,49], and species diversity shows a unimodal distribution pattern across altitude gradients [50].

Soil is another important determining factor of plant distribution. Soil texture, physicochemical properties, and microorganisms all affect vegetation distribution [51]. In this study, soil organic matter explained 13.0% and 14.0% of the β-diversity and its turnover component, respectively, and soil had a significant effect on community β-diversity and turnover but not on the nestedness component, as shown in Figure 4. The low explanation rate of soil factors relative to climate indicates that desert soil heterogeneity has a low influence on vegetation composition changes at a large scale, and this finding is similar to the results of a study conducted in the Gurbantunggut Desert [31].

For the relationship between species dispersal and geographic distance, we found that the species dissimilarity between communities increased with increasing the geographical distance. We found that species dissimilarity among communities became greater with increasing the geographical distance. The results showed that geographical patterns explained 3% and 1% of the β-diversity and its turnover component, respectively, which were much lower than the explanation rates of climate and soil factors, as shown in Table 2. This indicates that changes in the geographical patterns in this area have little effect on plant community assembly.

### 5.3. Roles of Environmental Filtering and Dispersal Limitation on Plant Community β-Diversity

**Figure 6:**
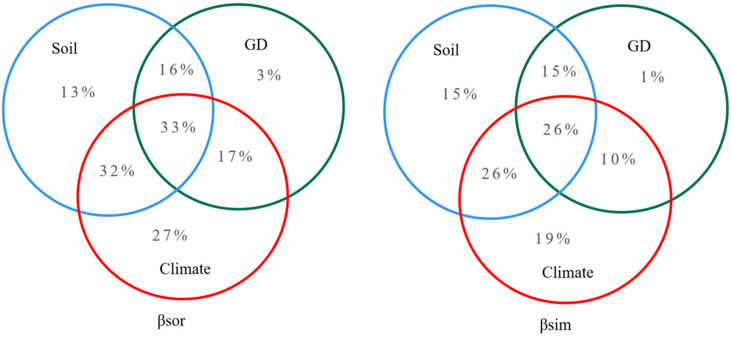
Analysis of the climatic, soil, and geographical distance on beta-diversity and its turnover component based on the MRM model.

Plant community β-diversity results from a combination of environmental filtering and dispersal limitation [52,53]. Niche theory and neutrality theory are widely accepted concepts for plant community assembly [54], and this study showed that habitat filtering and dispersal limitation determined the formation of the β-diversity patterns of the plant communities in the North Mountain and Maxian Mountain areas of Dunhuang. This result also indicated that environmental filtering, as described by climatic and soil factors, and dispersal limitation, as characterized by geographical patterns, were important drivers of plant community β-diversity and species turnover [55]. However, their effects on community β-diversity and its turnover component differed, with climate, soil, and geographical patterns individually explaining 27.0%, 13.5%, and 2.9% of the community β-diversity, respectively, and 19.0%, 15.0%, and 0.9% of its turnover component, respectively, as shown in Figure 6.

The effect of environmental filtering was much greater than that of diffusion limitation, and similar results have been obtained in β-diversity studies in other desert areas [56]. This indicates that the environmental filtering effect on β-diversity is significant in Dunhuang’s North Mountain and Maxian Mountain areas, where the environment is barren and the species structure is simple. We found that the denuded areas of the Maxian Mountains had thin soils and sparse species distributions, while, in the Qilian Mountains floodplain area, which has good soil texture and hydrothermal conditions, community species distributions were relatively dense. Climatic, soil, and geographical patterns together explained 33.0% of the community β-diversity in our study area, leaving 67.0% that could not be explained by these factors. These results indicated that unknown factors might exist, such as anthropogenic disturbance and study scale, and these may play an important role in affecting the β-diversity of community patterns in dry zones.

## 6. Conclusions

The role of environmental filtering and dispersal limitation on the plant community β-diversity was confirmed through our investigation of plant communities in the North Mountain and Maxian Mountain areas of Dunhuang. This study provides theoretical support for plant community assembly in this dry zone. As the differences in climatic factors and soil factors increased, the species dissimilarity indices of plant communities in Dunhuang increased accordingly.

The results of this study show that the β-diversity of plant communities and its turnover component in the Hexi Corridor were influenced by a combination of environmental filters and dispersal constraints. Environmental factors such as climate, soil, and altitude had a critical influence on β-diversity, with proportions of 27.0%, 13.5%, and 2.9%, respectively, while geographical patterns accounted for only 3.0%. Plant community turnover was more strongly influenced by environmental factors such as climate and soil at 19.0% and 15.0%, respectively, while geographical patterns accounted for only 1.0%. The environment was an important factor that influenced plant community construction and species turnover in this dry zone.

In the future, we should build upon this foundation and comprehensively consider factors such as climate, soil physicochemical properties, topography, and human disturbances to delve deeper into the influences of other unknown processes. This will enable a more accurate investigation of β-diversity in plant communities in the Gobi Desert.

## Figures and Tables

**Table 1:** Composition of families, genera, and species of vegetation in the research area.

NO.	Plant Families	Genus		Species	
		Quantity/pc	Percentage %	Quantity	Percentage %
1	Chenopodiaceae	12	26.70%	14	24.56%
2	Asteraceae	6	13.33%	12	21.05%
3	Poaceae	6	13.33%	7	12.28%
4	Fabaceae	4	8.89%	4	7.02%
5	Zygophyllaceae	3	6.67%	4	7.02%
6	Liliaceae	1	2.22%	3	5.26%
7	Polygonaceae	2	4.44%	2	3.52%
8	Brassicaceae	2	4.44%	2	3.52%
9	Tamaricaceae	2	4.44%	2	3.52%
10	Apocynaceae	1	2.22%	1	1.75%
11	Crassulaceae	1	2.22%	1	1.75%
12	White Flowering Danes	1	2.22%	1	1.75%
13	Ephedra	1	2.22%	1	1.75%
14	Buttercup family	1	2.22%	1	1.75%
15	Rosaceae	1	2.22%	1	1.75%
16	Caryophyllaceae	1	2.22%	1	1.75%

**Table 2:** Effects of climatic factors, soil factors, and geographical distance on β-diversity and turnover component in plant communities.

Influencing Factors	β-Diversity		Turnover	
	R^2^	*p*	R^2^	*p*
Climate factors	0.27	<0.001	0.19	<0.001
Soil factors	0.13	<0.001	0.15	<0.001
Geographical distance	0.03	<0.001	0.01	<0.001
Climate factor with soil factor	0.32	<0.001	0.26	<0.001
Climatic factors with geographical distance	0.17	<0.001	0.10	<0.001
Soil factor with Geographical distance	0.16	<0.001	0.15	<0.001
Climatic factor, Soil factor and Geographical distance	0.33	<0.001	0.26	<0.001

## Data Availability

All the data are available from the corresponding author on reasonable request.
